# Dr. William Pulteney Alison

**Published:** 1859-11

**Authors:** 


### NECROLOGICAL NOTICES.

THE death of WILLIAM PULTENEY ALISON has caused an irreparable void in the medical as well as social circle. Greatly distinguished as a physician, and everywhere recognized as a man of sterling Christian principles and untiring philanthropy, his loss will indeed be deeply mourned.

Dr. Alison was born in 1790, of highly intelligent and cultivated parents, his father being a popular preacher and author, and his mother a daughter of Dr. John Gregory. In his youth, Dr. Alison evinced decided military propensities, but in compliance with his father's earnest entreaties soon abandoned them, and began the study of medicine under his uncle, the celebrated Dr. James Gregory, who then occupied the Chair of Practice of Physic in the University of Edinburgh. In 1820 he accepted the professorship of Medical Jurisprudence in the University, and afterwards succeeded Dr. Andrew Duncan, as Professor of the

Institutes of Medicine. In 1831 he published a synopsis of these lectures under the title of "Outlines of Physiology." A few years after he was unanimously offered the Chair of Practice of Physic, which he occupied most satisfactorily until 1855, when he was obliged to resign it on account of the weakened state of his health, produced by epilepsy, by which he was attacked thirteen years ago, during a visit to the Infirmary. These attacks recurred at unequal periods, shattering his naturally strong constitution, and causing its complete overthrow on the 22d of September.

During the intervals of his terrible disease Dr. Alison read both medical and scientific works and wrote constantly. His writings are so various and of so diversified a nature, that it would be futile to undertake their enumeration. Among the most remarkable of them, however, are his essays on the "Contagion of Fever," and on "Vital Affinity." He also greatly ameliorated the social condition of the people by his "Observations on the Management of the Poor in Scotland." His medical writings display wonderful research and reflection, and his language is always elegant and forcible, a talent shared by his brother, Sir Archibald Alison, the distinguished historian. He contributed many valuable papers to the *Cyclopedia of Practical Medicine*, and is particularly known by his "Outlines of Pathology," and "Outlines of Physiology," before mentioned. In 1858 he was elected President of the British Medical Association, at its meeting in Edinburgh; and the warmth of his reception proved how universally he was loved and respected. Above the many intellectual gifts with which he was endowed, and his high literary attainments, will be remembered his unfailing benevolence and kind, generous heart. His whole life was devoted to his profession, and he allowed nothing to interfere with its duties, often sacrificing to them his domestic and scientific pursuits.

Dr. Alison's wife died many years ago, and he leaves no immediate family to mourn his loss; but the tidings of his death will cause deep regret in the profession both of Europe and of this country.

Dr. Alison's life of seventy years was one of arduous toil, and successful mental and physical labor, and to him may well be applied the saying of Horace,—

"Opere in longo fas est obrepere somnum."

---

Dr. E. H. Barton, late of New Orleans, died of disease of the heart, at Columbia, South Carolina, on the 20th of September. Dr. Barton was a gentleman of high distinction as a medical practitioner and as professor in the University of Louisiana, and was also well known for his scientific attainments, having contributed much useful information on the subject of yellow fever, especially in his report as Chairman of the Sanitary Commission of New Orleans. During the Mexican war he had the control of the hospital at Vera Cruz, where, from his earnest attention to the wounded soldiers, he commanded the respect and love of all who knew him.

